# Centralized Ambulance Destination Determination: A Retrospective Data Analysis to Determine Impact on EMS System Distribution, Surge Events, and Diversion Status

**DOI:** 10.5811/westjem.2021.8.53198

**Published:** 2021-10-26

**Authors:** Gurvijay Bains, Amelia Breyre, Ryan Seymour, Juan Carlos Montoy, John Brown, Mary Mercer, Chris Colwell

**Affiliations:** * University of California, San Francisco, Department of Emergency Medicine, San Francisco, California; † San Francisco Emergency Medical Services Agency, San Francisco, California

## Abstract

**Introduction:**

Emergency medical services (EMS) systems can become impacted by sudden surges that can occur throughout the day, as well as by natural disasters and the current pandemic. Because of this, emergency department crowding and ambulance “bunching,” or surges in ambulance-transported patients at receiving hospitals, can have a detrimental effect on patient care and financial implications for an EMS system. The Centralized Ambulance Destination Determination (CAD-D) project was initially created as a pilot project to look at the impact of an active, online base hospital physician and paramedic supervisor to direct patient destination and distribution, as a way to improve ambulance distribution, decrease surges at hospitals, and decrease diversion status.

**Methods:**

The project was initiated March 17, 2020, with a six-week baseline period; it had three additional study phases where the CAD-D was recommended (Phase 1), mandatory (Phase 2), and modified (Phase 3), respectively. We used coefficients of variation (CV) statistical analysis to measure the relative variability between datasets (eg, CAD-D phases), with a lower variation showing better and more even distribution across the different hospitals. We used analysis of co-variability for the CV to determine whether level loading was improved systemwide across the three phases against the baseline period. The primary outcomes of this study were the following: to determine the impact of ambulance distribution across a geographical area by using the CV; to determine whether there was a decrease in surge rates at the busiest hospital in this area; and the effects on diversion.

**Results:**

We calculated the CV of all ratios and used them as a measure of EMS patient distribution among hospitals. Mean CV was lower in Phase 2 as compared to baseline (1.56 vs 0.80 P < 0.05), and to baseline and Phase 3 (1.56 vs. 0.93, P <0.05). A lower CV indicates better distribution across more hospitals, instead of the EMS transports bunching at a few hospitals. Furthermore, the proportion of surge events was shown to be lower between baseline and Phase 1 (1.43 vs 0.77, P <0.05), baseline and Phase 2 (1.43 vs. 0.33, P < 0.05), and baseline and Phase 3 (1.43 vs 0.42, P < 0.05). Diversion was shown to increase over the system as a whole, despite decreased diversion rates at the busiest hospital in the system.

**Conclusion:**

In this retrospective study, we found that ambulance distribution increased across the system with the implementation of CAD-D, leading to better level loading. The surge rates decreased at some of the most impacted hospitals, while the rates of hospitals going on diversion paradoxically increased overall. Specifically, the results of this study showed that there was an improvement when comparing the CAD-D implementation vs the baseline period for both the ambulance distribution across the system (level loading/CV), and for surge events at three of the busiest hospitals in the system.

## INTRODUCTION

Ambulance distribution has been shown to have an impact on prehospital treatment and transport times and emergency department (ED) wait times, resulting in potential delays to care for time-sensitive medical conditions.^1^ Ambulance diversion has been shown to contribute to longer prehospital treatment/transport times, financial loss to hospitals, and increased ED crowding, and may be amenable to system-driven improvement.

The Centralized Ambulance Destination Determination (CAD-D, or CADDie) program was designed to manage the distribution of EMS patients throughout local hospitals to improve timely patient care. The CAD-D pilot project implemented an online, base hospital emergency physician and a paramedic supervisor to direct patient destination and distribution for stable, code 2 transport patients rather than have the destination chosen by each individual transporting paramedic. The physicians and the paramedic supervisor, using real-time and daily system data, provided real-time direction to EMS crews in the field to make transport-destination decisions. In assessing the program, our preliminary outcomes focused on transport per day to ED bed ratio, emergency medical services (EMS) surge events, and ambulance diversion, to determine whether there was improved distribution, and decreased surge and diversion rates. The city chosen to test this pilot project– San Francisco, California – encompasses 46 square miles and has a population of about 850,000 people. The population demographics are as follows: 46% Caucasian; 34% Asian; and 8% other, with a homeless population of roughly 8000.

## METHODS

**Figure 1:**
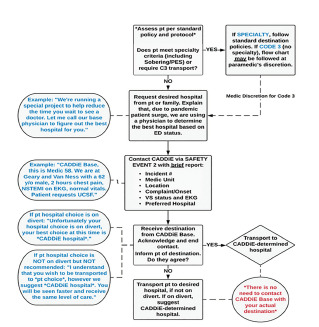
Flow Diagram with instructions on how to contact CADDie and how to assist with destination determination. *Pt*, patient; *PES*, Psychiatric Emergency Services; *CADDIe, c*entralized ambulance destination-determination; *ED*, emergency department; *NSTEMI*, non-ST-elevation myocardial infarction; *EKG*, electrocardiogram; *VS*, vital signs; *UCSF, University of California San Francisco.*

As part of the CAD-D protocol, paramedics called the EMS transport hub for instructions on where to transport patients if a non-emergent patient condition had been identified after paramedic assessment. The city has 11 EDs in the system, with one Level I trauma center. A paramedic supervisor paired with a base hospital physician were on duty during the study period to provide active direction/identification of destination for ambulances. Figure 1 shows the workflow for this pilot project, including details on when and how CAD-D was used.

**Population Health Research Capsule**

What do we already know about this issue?

*Emergency medical serivce systems are impacted by sudden surges that can occur throughout the day, as well as by natural disasters and the current pandemic.*

What was the research question?

*Can the use of an online base hospital physician and paramedic supervisor to direct patient destination and distribution decrease surges at hospitals?*

What was the major finding of the study?

*We found that there was improved distribution of patient transports, and that the average daily surge events decreased, while diversion rates steadily increased.*

How does this improve population health?

*Implementation could help offload busier hospitals and allocate resources appropriately to assist the most patients and spread distribution across a hospital system.*

Physicians were paired with paramedic supervisors to help facilitate an understanding of bed ratios and surge events that affected the EDs. The CAD-D destination recommendation used patient location, patient preference (if given), hospital diversion status, transport per hour-to-bed ratios, and patient chief complaint to assist the CAD-D paramedic and physician partner with the best hospital choice for the ambulance crew. Critically ill patients were transported to the geographically closest hospital appropriate to their medical condition (eg, trauma, stroke, ST-elevation myocardial infarction) without CAD-D contact. The standard EMS system Ambulance Destination Policy directed stable patients to be taken to the destination of their choice, if not on ambulance diversion or if the geographically closest facility was open to ambulance traffic.

This project was launched on March 17, 2020, when overall EMS call volumes were lower than normal due to the local coronavirus 2019 (COVID-19) surge and public health response, representing the baseline period. There were three phases of the project. In Phase 1 (April 16–July 2, 2020) CAD-D ambulance destination was a recommendation, and in Phase 2 (July 3 –October 26, 2020) CAD-D ambulance destination was a mandate. Based on an interim assessment of the data including system volume, patient distribution, compliance rates, and feedback from hospitals, a modified approach was attached to the CAD-D destination determination in an attempt to improve outcomes, thus creating the third and final phase. Phase 3 (October 27–February 2, 2021) was a hybrid system with CAD-D from 7 am to 12 am, coupled with a return to CAD-D destination as a recommendation. The analysis also included a baseline period prior to CAD-D institution.

We obtained data from existing datasets used for prehospital patient management: ReddiNet* ambulance diversion reports (a service of the Hospital Association of Southern California, Los Angeles, CA) and First Watch** (Carlsbad, CA) CAD-D data. ReddiNet is a web-based emergency medical communications system used to report hospital, patient, and emergency event status, and First Watch is a web-based service to improve operations, performance, clinical measures and provide early warning for crucial events.

The baseline period was the 30 days between March 17–April 15, 2020, coinciding with the day the local shelter-in-place order was issued until the start of the CAD-D program. Phase 1 had CAD-D operational 24 hours per day, and hospital direction to EMS crews was a recommendation. Phase 2 had CAD-D operational 24 hours per day, and hospital direction to EMS crews was mandatory. During Phase 3, CAD-D was operational between 7 am and midnight (hours during which six or more calls per hour are generated in the system), seven days per week, and hospital direction to EMS crews was a recommendation except for the busiest hospital in the system, where the destination determination (to or away from) remained mandatory.

In analyzing the data we used the coefficient of variation (CV) to measure the dispersion of data points about the mean, specifically by representing the ratio of the standard deviation to the mean. The ratio enabled us to measure relative variability between different datasets (eg, CAD-D phases), even if their means were different. This is important because the goal of CAD-D is to reduce variability in EMS patient distribution, relative to each hospital’s ED bed count, regardless of the average transport-to-bed ratio. This study looked at a new measure to determine whether the mean transport-to-bed ratio was significantly different between CAD-D phases (eg, fluctuations in call volume, potentially COVID-related) and whether the measure of variability between these phases would still be comparable using CV.

We used analysis of co-variability to compare mean CV across phases while controlling for total EMS volume, thereby helping us ensure that the differences seen in mean CV were not attributable to transport volume. We also made pairwise comparisons between phases, having controlled for total EMS transport and adjusted using Tukey’s methodology.

Over the course of the study, we collected data regarding the impact of CAD-D on the transports per hour: bed ratio in the EDs; the analysis of surge events; and the impact of this pilot project on diversion. Specifically, 56,684 EMS transports resulted from 911 calls in the city of San Francisco during the time of this project. Of the total number of transports, 40,365 (71%) were routed through CAD-D. Of the total number of CAD-D calls, 32,152 (80%) were logged with a valid incident number (unique call identifier) and a non-blank “requested hospital” field. Both of these were necessary to determine whether CAD-D had an impact on the outcome of the transport.

Valid entries in the log were joined with EMS transport data and categorized as follows: non-candidate, ie, the requested hospital from EMS matches the hospital recommendation from CAD-D; and candidate, ie, either CAD-D indicated in the “requested hospital” field that “no preference” was given, or the actual destination hospital did not match the requested hospital. In these cases, CAD-D may have influenced the destination of the patient. Of the total number of validated CAD-D calls, 6527 (20%) were CAD-D candidates. CAD-D candidate transports were classified as “positive impact” if the actual destination matched the recommended destination given by CAD-D. In other words, if EMS was directed to a hospital by CAD-D when they requested a different destination, or did not have a requested destination, CAD-D had an impact on the transport outcome. Of the validated CAD-D calls, 5559 (17%) transports were impacted by CAD-D.

### Outcomes

There were three primary outcomes: the ratio of EMS transports per hour to ED beds, EMS surge values, and ambulance mean time on diversion per day. The EMS transport per day-to-bed ratio was defined as the number of EMS transports to a hospital in a single day, in relation to the total number of licensed beds in that hospital’s ED. The CAD-D’s targeted max ratio, both daily and average, is 1.0, (ie, one EMS patient transported per 24 hours per licensed bed.) A lower CV indicated less relative variation in EMS transports and more even patient distribution, or “level loading.”

**Figure 2:**
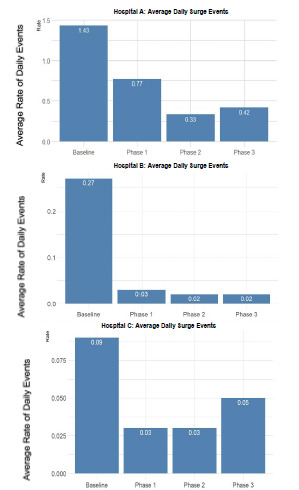
Comparing the average daily rate of surge events, in the different phases of the Centralized Ambulance Destination Diversion (CAD-D) project. Hospitals A, B and C showed decreased surge events in the CAD-D phases compared to baseline.

A surge event was defined as occurring when the number of ambulance arrivals to an ED in a given hour exceeded 30% of its licensed ED bed count or was **≥** 6. This was chosen because most hospitals in our system have a single ambulance-triage entry point that at maximum can process one stable EMS patient arrival per 10 minutes. “Hospital A” was chosen due to having the highest rate of surge events in the county (2020), and the highest rate of diversion among all hospital in that county (2020). Being the county’s only trauma center, Hospital A is also a specialty center for other types of critical patients (eg, stroke, STEMI); therefore, receiving a large number of Code 3 ambulances whose destination determination is unaffected by the CAD-D program. Hospital B and Hospital C were chosen as two of the other busiest hospitals in our system for comparison, and their data are also shown below in Figure 2. They are also specialty centers and receive Code 3 ambulances from the EMS system.

We also studied ambulance diversion to determine whether EMS transports affected rates of diversion as a system. Emergency departments went on diversion, meaning they would prefer EMS to transport their Code 2 transports to less impacted hospitals. In our data, the diversion rates for the system as a whole increased, which is discussed below.

## RESULTS

**Figure 3:**
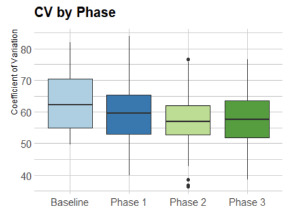
The coefficient of variation* among the baseline, Phase 1, Phase 2, and Phase 3 portions of the project. *Lower coefficient of variation in all phases compared to baseline showed improved level loading of the system with improved patient transport distribution. *CV*, coefficient of variation.

For each day, the CV of all ratios were calculated and used as a measure of EMS patient distribution among hospitals as seen in Figure 3, which presents the CV of all ratios. Mean CV was lower in Phase 2 as compared to baseline (1.56 vs 0.80, *P* = 0.002), and baseline and Phase 3 (1.56 vs 0.93, *P* = 0.007). This showed the optimal (smallest) variation occurred during the recommendation in phase 2, even over phases 1 and 3. A lower variation meant more appropriate level loading of the system. This may indicate that CAD-D as a mandate improved distribution over the system as a whole.

We used analysis of co-variability to compare mean CV across phases while controlling for total EMS volume, showing that the differences seen in mean CV were not attributable to transport volume. A global F-test was performed to determine whether at least two of the groups had underlying means that were significantly different after controlling for transport volume. There is significant evidence at the level that there was a difference in CV between at least two phases, after controlling for total EMS transport volume.

Figure 2 shows the results of the average daily rate of surge events. The results showed that compared to baseline vs phases 1, 2, and 3, there was a statistically significant difference demonstrating that the CAD-D project had a positive impact on the surge events at three of the busiest hospitals in the system. The proportion of surge events was lower between baseline and Phase 1 (1.43 vs 0.77. *P* = 0.002), baseline and Phase 2 (1.43 vs. 0.33, *P* < 0.00001), and baseline and Phase 3 (1.43 vs 0.42, *P* < 0.00001). The percentage of hours in which a surge event occurred was 46.1% lower in Phase 1 vs baseline, 76.9% lower in Phase 2 vs baseline, and 70.9% lower in Phase 3 vs baseline. The average daily rate of surge events was also studied in two other highly impacted hospitals in the system, Hospital B, and Hospital C.

**Figure 4:**
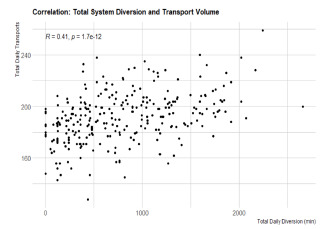
Daily system diversion totals from implementation of the study to January 2021. Total daily diversion on X-axis compared to total daily transports on y-axis. Total diversion time does not seem to correlate with daily transports.

Finally, CAD-D did not seem to decrease the ambulance diversion rates across the system as a whole in San Francisco County (Figure 4.) This could have been due to higher acuity of patients during the COVID-19 era, more inpatient admissions, more ED boarding, or other non-EMS-related conditions that contributed to less ability for hospitals to be able to handle EMS calls.

## DISCUSSION

When EDs become crowded, incoming ambulances are diverted to other hospitals to help ease this crowding. In 2003, 45% of United States EDs reported being “on diversion” at some point of the year. Common problems associated with diversion include prolonged transport times, delays in care, increased mortality, and lower hospital revenue.^12^ A systematic review from 2013 showed that smoothing elective surgery scheduling, adding ED fast tracks for inpatient boarders, and implementing regional cooperative agreements among hospitals are promising avenues for reducing diversion.^13^ However, diversion continues to be an issue, prompting the creation of a potential solution.

The CAD-D pilot program has shown that the implementation of a physician and paramedic supervisor joint destination center to monitor and divert ambulances to less impacted hospitals as a way to level load the system had mixed effects. In our urban EMS system, the large, tertiary care hospitals frequently became the most impacted. To better offload these few hospitals and better distribute the EMS transports across the 11 hospitals in this system, the CAD-D project helped with distributing EMS transports to other hospitals. The CV is a marker for this distribution, with a lower number indicating that there were more patients spread across the different hospitals instead of all going to the few heavily impacted hospitals. However, when looking at heavily impacted hospitals in the system prior to CAD-D, the average daily surge events seemed to have decreased at Hospital A with statistical significance. Finally, ambulance diversion steadily increased across the system as a whole throughout the different phases. This finding could mean that although daily surge events decreased across the busiest hospitals, this had no bearing on whether a hospital went on diversion. It could also show that EMS transports have no bearing on whether a hospital goes on ambulance diversion. This could be due to higher acuity of patients during the COVID-19 era, more inpatient admissions, more ED boarding, or other non-EMS-related conditions that contributed to less ability by hospitals to handle EMS calls. However, interpretation of this preliminary data is challenged by many unaccounted factors, most notably the effect of a COVID-19 surge on the EMS system.

## LIMITATIONS

One of the limitations of this study is that it was approved for and implemented during the COVID-19 pandemic. Because of this, the overall patient volumes were lower during the baseline period and all three phases. The decrease in patient volumes during the pandemic was difficult to foresee, but the overall phases of this study had similar patient volumes, as all were during the lockdown of the city. This study was also limited by lack of full compliance of all ambulance services in the county. While two of the ambulance services more consistently contacted the paramedic and physician supervisors, this was not the case for all services involved. More research is needed to determine the exact percentages of non-compliance and effect on this pilot study. Furthermore, at times there was a discrepancy between which hospital the ambulance was recommended to attend to and where the patient was transported. Within the context of the COVID-19 pandemic, and decreased transport volumes, this pilot project may prove to be more beneficial when surge events become more prominent as patient volumes return to the EDs.

## CONCLUSION

In this retrospective review of a novel ambulance distribution system, we found that there was improved distribution of patient transports, and that the average daily surge events decreased at three heavily impacted hospitals in San Francisco County. Interestingly, diversion rates steadily increased as a whole. Since the diversion rate seemed to steadily increase during all phases of CAD-D implementation including the baseline phase, along with overall increase in EMS call volume, it may be a consideration that EMS and prehospital patient arrival has no bearing on the use of ambulance diversion by hospitals.
